# Acute Mastoiditis—Early Operation a Necessity for the Patient’s Safety*Read at annual meeting of the Medical Association of the Southwest at Kansas City, Mo., October, 1908.

**Published:** 1909-02

**Authors:** Robert E. Moss

**Affiliations:** San Antonio, Texas


For Texas Medical Journal.

### Acute Mastoiditis—Early Operation a Necessity for the Patient's Safety.*

BY ROBERT E. MOSS, M. D., SAN ANTONIO, TEXAS.

I felt very much complimented when requested to read a paper before this body on the above subject. It is one that has been written about so frequently in the last few years by many men of exceptional ability and their published articles, easily obtained, if not already familiar to everyone, that I feel a hesitancy in appearing before you. As one of my former instructors was fond of saying, however, that "line by line and precept upon precept we acquire useful knowledge."

This is an interesting subject to study its history, and to think how old, and yet new, it is. The first mastoid operation done to relieve a suppurative condition was performed about 1760, by Louis Petit, of France. The operation was not favorably received by the profession, but generally condemned, and very little done until recent years. I presume Schwartze deserves more credit than any other man for his masterly work in establishing the operation on a firm basis. There are many brilliant men in our own country who have done much to work out details and perfect the present technique. It is very surprising in this day of suc-

*Read at annual meeting of the Medical Association of the Southwest at Kansas City, Mo., October, 1908.

cessful surgery to think that any one could be found who would condemn the operation, or advise waiting, whilst using palliative measures until there were positive danger symptoms, and yet I presume such is the case everywhere in this country. It is not confined to the general practitioner, either, by any means, but we find men of repute in our own ranks who advise the ice coil, leeches, blisters, poultices, Wilde's incision and even Antiphlogistine. To quote from one of our recent authors, "We may affirm that whenever a Wilde's incision is indicated, a mastoid is imperative." The question has constantly occurred to me in connection with this operation, why the difference on the part of patient and the average general practitioner in regard to this, as compared to that for appendicitis? Are we to blame? I heard a prominent general surgeon in one of our large cities of the Middle West say, only a few year ago, when assisting me with a mastoid operation on one of his patients who was visiting in my city at the time, that he proposed in future to do his own mastoid operations, because the specialists of his city were not thorough enough. There is a phrase in the Bible which says: "Many are called, but few are chosen." How true when applied to surgery; there are many operators, but few surgeons. It would be interesting to know what percentage of chronic suppurative cases began with distinct mastoid symptoms.

Heath says the antrum is involved in every chronic case. If this was primary, it would be another point in favor of early operation. There is no question but that patients with well-marked cases of mastoiditis, just as there are with appendicitis, get well without operation, but who among the many surgeons of world-wide fame can predict the outcome of any case of appendicitis? It is equally true of mastoiditis. I have come to the very positive decision, after many years of study, careful observation and about two hundred operations that, when we are sure of our diagnosis, we should advise an operation. The question of diagnosis should not be difficult, and yet there are cases where many of the classical symptoms are lacking, and we have wide destruction of the cancellated structure of the mastoid; also cases where a furuncle will give us all the symptoms of a mastoiditis. I have seen a few cases of aspergilus where the patient came under observation after a week or more that caused me to be in doubt. The history of the case with a careful analysis of the symptoms at the time makes the diagnosis a comparatively easy one. There are cases where there is no discharge from the ear, only slight pain, temperature normal, scarcely any swelling, but little tenderness on pressure, and yet the entire mastoid be involved. I have had two such patients. One refused an operation for months; the other was sent to me from another town, after six weeks of treatment for malaria, rheumatism, etc. His physicians were good men, too, but had overlooked the ear entirely, as there were no very positive symptoms. I came very near operating once on a young lady; in fact, had arranged to operate the next day, but went back late in the afternoon and found a discharge of pus and my patient perfectly comfortable; it was a furuncle. This came on during an attack of grippe, and I assure you it looked like a typical mastoiditis. Our text-books tell us that no error need occur, if, when making pressure, we are careful to press backwards and inwards just behind the insertion of auricle. This manipulation does not move the fibro-cartilaginous canal; therefore, if pain is elicited, it is due to mastoid inflammation, and not the external canal. The case to which I have just alluded proved an exception. Of course, where there is the usual condition of things, no doubt arises at all as to diagnosis, and it is simply a question to operate or not to operate. The discharge should be examined microscopically, and if the more virulent germs, such as the pneumococcus streptococcus, streptococcus capsulatus, are found, we should insist upon immediate operation.

In measles, diphtheria, scarlet fever and pneumonia, we should operate at once if there is the slightest evidence of involvement of the mastoid. One of the few patients I have lost from acute mastoiditis was a delayed case complicating scarlet fever, where there was a brain abscess and sinus thrombosis. Where there is a chronic otorrhea and acute symptoms occur, with pain and tenderness over mastoid, we should operate at once. These cases give us a high death rate as compared to those of the first attack. They seem to be followed by brain abscess, sinus thrombosis and meningitis more frequently. The death rate following this class of cases is placed as high as 20 to 25 per cent. Just why anyone should advise delay when the diagnosis is made, I can not understand. The early operation is so simple and safe, and the convalescence so satisfactory as compared to delayed cases. The same can be said of appendicitis, in early operations where there are no complications, in the hands of skillful surgeons, there is practically a hundred per cent of recoveries, whilst in delayed cases the same men have from 10 to 20 per cent of fatalities. It is the skillful men in the surgical world who are pleading for early operations. Our first duty is to our patient, and his welfare to be considered, and our own reputations second. It does not matter, however, how skillful or thorough our operation, if the patient dies there are many ready to condemn and cast doubts upon surgeons and surgery generally. It is the number who die from delayed operations, and at the hands of indifferent operators, that causes a great many to postpone and debate the question whether their chances would not be better without an operation. I do not know whether I have seen more complicated cases than the average man, but I wish to assure you that I am never called to see one that I do not dread it, and wish the other fellow had the case. If it was possible for us to say this case will recover or that one must be operated on, how satisfactory it would be, but that is impossible. *I am very positive we can not rely upon symptoms to guide us.*

My first case of sinus thrombosis was a delayed case; the patient going around and doing her work until she had the chill caused by an infected sinus. I feel very sure in saying that if every case of mastoiditis was operated upon as soon as the diagnosis was made, by men who were skillful operators, that it would not be many years before patients would willingly consent to the operation. Our statistics would be so convincing that no arguments would be necessary.

---

FORT WORTH, TEXAS, January 22, 1909.

*Dr. F. E. Daniel, Chairman of the Section on State Medicine and Public Hygiene, Austin, Texas.*

DEAR DOCTOR: I wish to notify you, as is the regular custom, that the program of your Section must be printed in the *State Journal of Medicine* appearing one month before our annual meeting; that is, in the April *Journal.* This will require your program to be in the State office by March 20th, and it would be well for you to state to contributors that they must have the program in your hands by March 15th.

According to the Constitution, all papers from Texas physicians must have been read before local county societies, be typewritten, and capable of being read in twenty minutes.

I trust you will immediately co-operate with Dr. L. B. Bibb, Secretary of your Section, and have the program in my hands by the above date.

With kind regards and best wishes.

Very sincerely yours,

I. C. CHASE.

